# Labeling food allergens in the packaged food pyramid groups in Brazil: analysis of descriptions, ambiguities, and risks

**DOI:** 10.1590/1984-0462/2022/40/2021079IN

**Published:** 2022-06-10

**Authors:** Joice Ferreira Lopes, Mary de Assis Carvalho, Nilton Carlos Machado

**Affiliations:** 1 Universidade Estadual Paulista “Júlio de Mesquita Filho”, Botucatu, SP, Brazil.

**Keywords:** Allergens, Food hypersensitivity, Industrialized foods, Food labeling, Alérgenos, Hipersensibilidade alimentar, Alimentos industrializados, Rotulagem de alimentos

## Abstract

**Objective:**

The aim of this study was to evaluate allergenic labeling components of packaged foods for “What is the quality of food labels?” and “What is the group of Brazilian Food Pyramid that ‘May contain’ is predominant?.”

**Methods:**

The photographs of 916 products were obtained, of which 518 were analyzed. Data from each label were evaluated according to Brazilian Food Pyramid Groups (i.e., Cereals, Fruits, & Vegetables; Soybean & products; Milk & dairy products; Meat & eggs; Fats & oils; and Sugars & sweets). Ten items were analyzed in each label, namely, the presence of a list of ingredients, alert phrase for allergy sufferers, grouping of the alert phrase, phrase location, uppercase phrase, the phrase in bold, the color of alert phrase contrasting to the background, adequate font size, do not claim the absence for any allergen with the ingredients, and others factors that make it difficult to read. For the second question, a structured questionnaire was completed, and products were classified into two categories, namely, “Contain” and “May contain.”

**Results:**

The quality of the label was appropriate, and 69% of packaged foods had at least one allergen. The information “May contain” were higher in cow’s milk (Cereals and Meat & eggs), soy (Soybean & products), and egg protein (Cereals). Soybean & products were the highest insecurity group.

**Conclusions:**

Brazilian health professionals can count on good-quality labeling of packaged products. Consequently, they could promote patients’ and parents/caregivers’ education to consult the labels and manage the risks in processed foods about precautionary allergen labeling. Soybean & products were the most significant insecurity for food choices between Brazilian Pyramid Groups.

## RESUMO

**Objetivo:**

Avaliar os componentes alergênicos da rotulagem de alimentos embalados e responder “Qual é a qualidade da rotulagem?” e “Qual é o grupo de alimentos em que a expressão ‘Pode conter’ é predominante?”.

**Métodos:**

Foram obtidas fotografias de 916 produtos, dos quais 518 foram analisados. Os dados dos rótulos foram avaliados de acordo com os grupos da pirâmide alimentar brasileira (Cereais, Frutas & Vegetais; Soja & Derivados; Leite & Laticínios; Carne & Ovos; Gorduras & Óleos; Açúcares & Doces). Dez itens foram analisados nos rótulos: presença de lista de ingredientes; frase de alerta para alérgicos; frase de alerta; local da frase; frase em letras maiúsculas; frase em negrito; a cor da frase de alerta contrastando com o fundo; tamanho de fonte adequado; não alegar ausência de qualquer alérgeno; outros fatores de difícil leitura. Para a segunda questão, foi respondido questionário estruturado, e os produtos classificados em categorias: “Contém” e “Pode conter”.

**Resultados:**

A qualidade do rótulo foi adequada e 69% dos alimentos embalados continham pelo menos um alérgeno. A informação “Pode conter” foi mais identificada em: proteína do leite (Cereais e Carnes e ovos), soja (Soja & derivados) e proteína do ovo (Cereais). Soja e derivados foi o grupo alimentar de maior insegurança.

**Conclusões:**

Profissionais de saúde podem contar com rotulagem de boa qualidade dos produtos embalados e, consequentemente, promover a educação de pais/cuidadores para consultar os rótulos e gerenciar os riscos em alimentos processados. Soja e produtos são os alimentos com maior insegurança entre os Grupos da Pirâmide Brasileira.

## INTRODUCTION

Food allergy (FA) is characterized by individual susceptibility to specific foods that occur reproducibly and safe to most healthy individuals.^1–4^ The prevalence estimated that approximately 5% of adults and 8% of children have FAs.^5^ It is estimated that over 220 million people worldwide suffer from some form of FA.^6,7^ Certainly, FA is a major global health problem, and the geographical variability distribution can be linked to genetic, environmental, and lifestyle habits.^8^ The list of foods implicated varies depending on the country.^9^ While more than 160 foods can cause allergic reactions, eight foods are significant allergens (such as cow’s milk, soy, eggs, wheat, fish and crustacean shellfish, peanuts and nuts). These food allergens are responsible for 90% of food reactions, particularly in children within the first year of life. Optimistically, in over 80% of cases, these allergies recover spontaneously within the first 3 years of life.^10^ Clinical management of FA includes allergic symptom recognition and prompt treatment.^11,12^ Regardless of novel therapies, avoidance allergens are imperative.^1,6,13,14^

In addition, the world is experiencing the highest rates of diet-related chronic diseases, and there is an excellent emphasis on consumers making healthier food choices. Considering this, allergenic foods’ labeling is an essential public health measure to help vulnerable consumers avoid clinical reactions. Therefore, the declaration of ingredients on the packaged food label is an essential source of information for consumers. Errors in the process of reading and interpreting labels can generate potential risks for people with FAs. Education is particularly needed, and proper labeling and reading are crucial to the success of avoidance diets, given that misinterpretation of food labels is a common cause of accidental ingestion.^15,16^

Unfortunately, a hidden allergen that does not appear on the label may occur during production or manufacturing secondary to cross-contamination. Precautionary allergen labeling (PAL), for example, “May contain,” is present on many commercial food labels warning consumers that an allergen may have occurred during manufacturing. However, the use of PAL is voluntary and unregulated in many countries. In Brazil, the National Agency of Sanitary Surveillance^17^ has established requirements for labeling the main foods that cause FAs. Given all concerns manifest, this study aimed to evaluate Brazilian packaged foods’ labeling for allergenic components. The first goal was to qualify the food labels regarding allergenic components presented on the packaged foods after implementing mandatory labeling.^17^ Therefore, the first question being addressed is, “What is the quality of food labels?.” The second goal was to identify the presence of allergens in foods (Contain) and the PAL (May contain). The second question being addressed is, “What is the group of Brazilian Food Pyramid that PAL is predominant? This study hypothesized that the description of food allergens in packaged foods is fulfilled by the food industries, being transparent and safe for allergic consumers.

## METHOD

This is an observational, cross-sectional study that evaluated a convenience sample of Brazilian Food Pyramid Groups of packaged food labels, available in three supermarkets of different chains in Araçatuba (São Paulo State). Data were collected between August and September 2018, avoiding variations in the labeling of the evaluated foods.

Regarding the inclusion criteria, in the first step, the principal researcher searched for different products; collected foodstuff, fancy name/brand, and available versions (e.g., different flavors); and elaborated a list. The sample for analysis was obtained, consisting of a minimum of 50% of packaged food brands of each foodstuff of the initial list. Products were randomly selected with as many manufacturers as possible to obtain a broad representation. The foods included were not only for use in the pediatric age group. In the second step, two digital photographs were taken for each product in all its dimensions (i.e., central panel, sides, and undersides). Each product was verified to ensure the data entered for the product and the accompanying photos are correct.^18^

An initial database of 916 packaged products was arranged. After an initial review, same products were excluded if the identical products were the same bit with different sizes or the photographs were not clear. Thus, 518 packaged foods remained for analysis. Data from each label were entered into an Excel spreadsheet according to categories of the Brazilian Food Pyramid Groups (i.e., Cereals, Fruits & Vegetables; Leguminous – Soybean & products; Milk & dairy products; Meat & eggs; Fats & oils; and Sugars & sweets).

The labeling characteristics were evaluated in terms of the description of allergenic components considered mandatory by the labeling control agency in Brazil.^17^ For the answer to the first question, “What is the quality of food labels?,” 10 items were analyzed, namely (1) the presence of a list of ingredients and sales denomination, (2) alert phrase for allergy sufferers, (3) grouping of the alert phrase, (4) phrase location after the ingredients, (5) uppercase phrase, (6) phrase in bold, (7) color of alert phrase contrasting to background, (8) adequate font size, (9) do not claim the absence for any allergen with the ingredients, and (10) other factors that make it difficult to read the information (phrase covered by the fold, labels, twisted). Each item that is considered adequate is given one point. The sum was considered labeling quality and presented in values ranging from 0 to 10 for each Brazilian Food Pyramid Group.

For the second question’s answer, a structured questionnaire was completed for each product after reviewing the label. The photographs were evaluated on data relevant to allergy sufferers, such as the product’s name, nutritional information, list of ingredients, and allergy alert. In the analysis of food labels, products were classified according to Brazilian Food Pyramid groups in two categories: (1) Mandatory, “Contain” when the allergen is added as an ingredient And (2) PAL “May contain” when the allergen might be inadvertently in the food because of cross-contact. Considering the prevalence, special analysis with cow’s milk, soy, and egg protein allergy will be considered.

The different designations described in this text were as follows:

- **Food allergens:** components of foods that trigger immunological reactions and initiate the development of an FA.
- **Nutrition labeling:** is any inscription, legend, image or any descriptive, or graphic material written, printed to help consumers select healthy diets that meet dietary recommendations.
- **PAL:** information destined to consumers with FA about a significant risk of reacting to a product.
- **Food category:** foods, according to the type of food product, such as meals, dishes, or individual food items such as Cereals.
- **Food packaging:** includes commercially processed and packaged food products that protect and preserve the product, providing consumers with product information, including ingredient and nutrient content.

The analysis was performed using GraphPad Prism version 8.4.0 for Windows (GraphPad Software, San Diego, CA, USA; www.graphpad.com). Continuous variables were expressed as a mean and standard deviation. The categorical data were reported in count and percentage and analyzed using Fisher’s exact test. One-way analysis of variance, followed by Tukey’s multiple comparisons test, was used to compare the quality of the label scores. All statistical testing used a significance level of p<05. This study was approved by the Research Ethics Committee of the Botucatu Medical School (CAAE. 94086718.4.0000.5411).

## RESULTS

**Figure 1:**
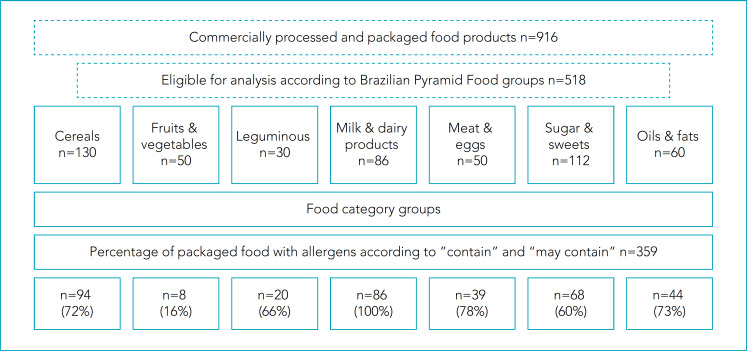
Percentage of packaged foods with allergens in the different groups of the Brazilian food pyramid.

Figure 1 presents the percentage of 518 packaged foods evaluated, of which 359 (69%) had at least one of eight main allergens. The percentage of foods allergens was 100% for Milk & dairy products. The other groups had more than 60% of the food allergens, except for Fruits/vegetables which has a lower percentage (16%).

The quality of the label of the packaged foods was appropriate in all 10 items analyzed. The percentage of adequacy for each item of the quality of the labeling was Items 1 (100%), 2 (99%), 3 (99%), 4 (97%), 5 (98%), 6 (98%), 7 (97%), 8 (98%), 9 (97%), and 10 (95%). Consequently, the values were close to the maximum in all groups of Brazilian Food Pyramid: Cereals (9.2), Fruits & Vegetables (9.8), Leguminous – Soybean & products (9.4), Milk & dairy products (9.6), Meat & eggs (9.6) Fats & oils (10.0), and Sugars & sweets (9.8). There was no statistical difference between the groups.

**Figure 2:**
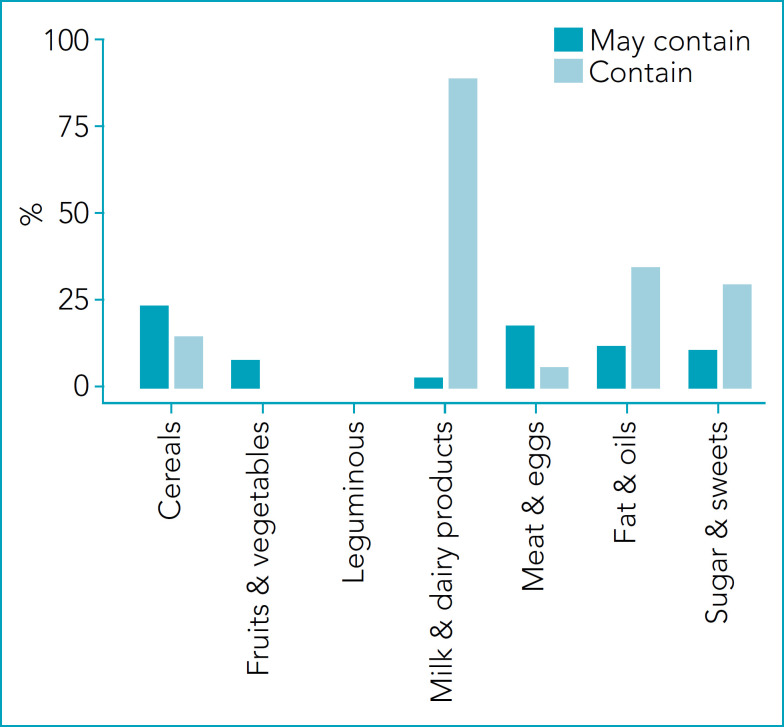
Percentage of foods that offer risk of reactions to children with cow’s milk protein allergy in the different groups of the Brazilian food pyramid.

**Figure 3:**
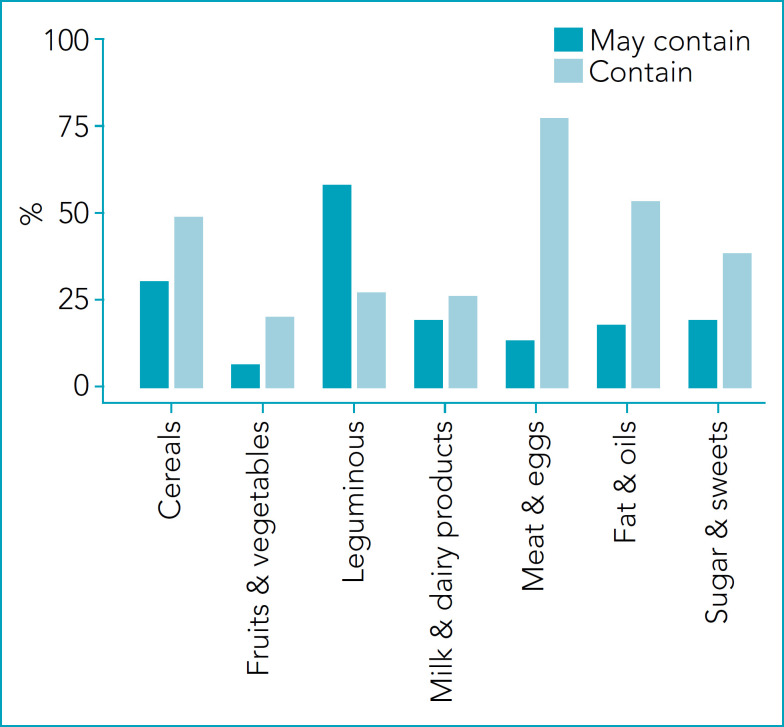
Percentage of foods that offer risk of reactions to children with soy protein allergy in the different groups of the Brazilian food pyramid.

**Figure 4:**
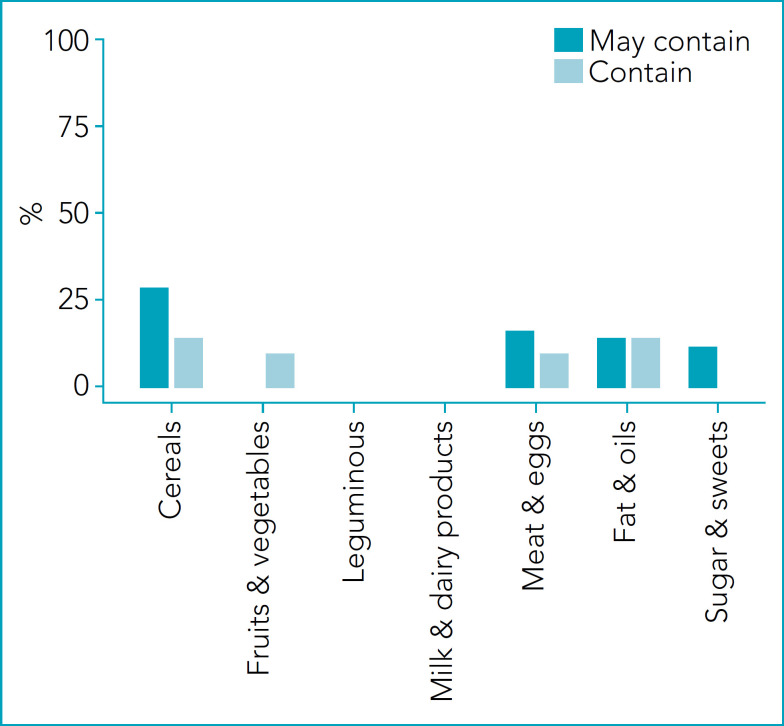
Percentage of foods that offer risk of reactions to children with egg protein allergy in the different groups of the Brazilian food pyramid.

Figure 2 presents the proportion of foods with the risk of reactions in children with cow’s milk protein allergy. The proportion of “Contain” is higher in Milk & dairy products, followed by Fats & oils and Sugars & sweets. The proportion of “May contain” is higher in Cereals and Meat & eggs. There is no risk for Fruits/Vegetables. Figure 3 presents the proportion of foods with the risk of reactions in children with soy protein allergy. The proportion of “Contain” is higher in Meat/eggs (64%). In addition, it is present in more than 30% of Cereals, Fats & oils, and Sugars & sweets. The proportion of “May contain” is higher in Leguminous (Soybean & products=47%) and Cereals (23%). The lower value was observed in Fruits & Vegetables (2%). Figure 4 presents the proportion of foods with the risk of reactions in children with egg proteins allergy. The egg was present in lower proportions (<10%) in all groups. The proportion of “May contain” is higher in Cereals (21%). There is no risk for Fruits & Vegetables and Sugars & sweets.

**Figure 5:**
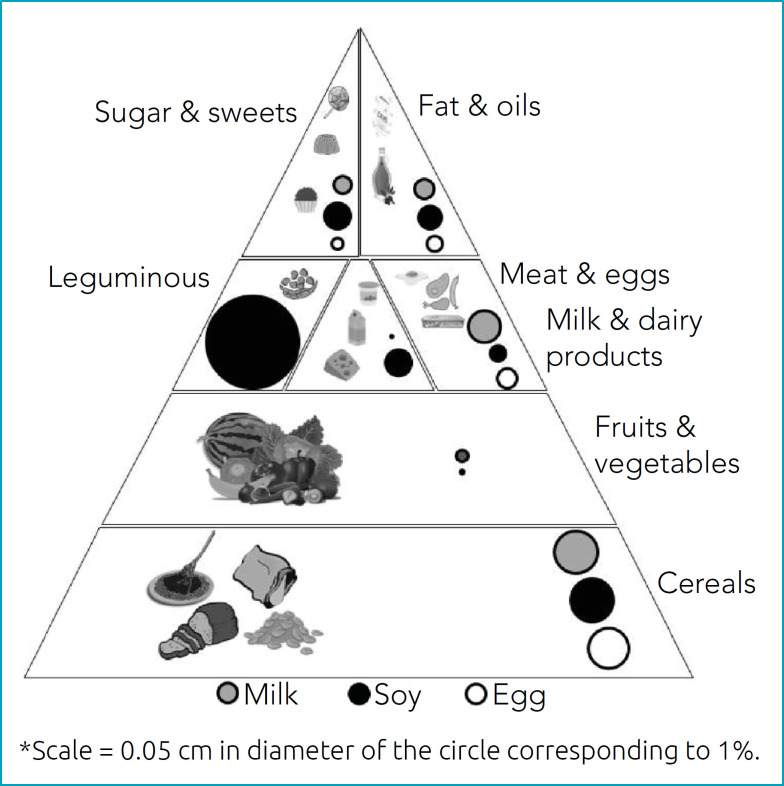
Brazilian food pyramid and the proportion of foods that offer risk of reactions “May contain” to children with food allergy.

Figure 5 shows the proportion of “May contain” as an alert for children with FA regarding the presence of milk, soy, and eggs in Brazilian Food Pyramid Groups. Soybean & products were the highest insecurity group; once the information “May contain” for this allergen, it is identified in all food groups. Fruits & vegetables were the highest security group for food choices. The other groups present different proportions of risk for the three allergens. Cereals present a similar proportion of “May contain” for milk, soy, and egg. Sugar & sweets and Oils & fats groups had similar risk for soy, milk, and egg.

## DISCUSSION

This study evidenced that the Brazilian food industry’s current practice in describing the allergens present in packaged foods was of high quality. The Labeling Quality Score was applied concerning the allergic public’s information, those packaged foods that contained description in alert phrases, or the ingredients for some food allergen required by the Brazilian labeling resolution for possible allergy-causing foods. The labeling in Brazil is presented with quality and is under the criteria for labeling allergens. Thus, it is confirmed the applicability of the Brazilian resolution requirements demonstrated by the high values of the Labeling Quality Score for all food groups evaluated in this study. Thus, after 5 years of ANVISA regulations,^17^ it is possible to consider that patients have benefited from the resolution.

Food labels are a source of product information for patients and parents/caregivers of children with FA. However, few studies on the use of food labels have been published. Consequently, comparisons are difficult because different methodologies have been used. Several characteristics of the label were classified as severe or very serious problems by about 40% of respondents with FA.^19,20^ Joshi et al.^19^ studied the accuracy with which parents of children with FAs could identify allergenic foods on food labels. They found that many parents could make mistakes when reading food labels. Milk & dairy products were the most difficult for parents to identify; only 7% of parents of children with milk allergies correctly identified all the names on the labels that declared milk. However, most parents were able to identify wheat or egg on labels correctly. They also noted that almost 50% of children with FAs considered it necessary to contact manufacturers to determine whether an allergenic food is present in a product. In this study, the findings showed that one or more of eight allergen ingredients in packaged foods sold in Brazil are high (69%). Milk & dairy products were the groups with highest risk. Fruit and Vegetables offer the lowest risk of the presence of food allergen.

Pieretti et al.^18^ found 17% of products containing warning labels for allergy sufferers. The most frequently listed allergens on the study labels were walnuts (61%) and peanuts (48%). In this study, 69% of the sample contained a warning for allergy patients, highlighting soy, cow’s milk, and wheat, among the categories of foods evaluated.

Current treatment of FA involves the primary exclusion of allergenic foods and their derivatives. To achieve this goal, the information on the packaged food labels must be clear and accurate. Allen et al.^21^ mentioned different risks for contamination of the packaged product, such as auxiliary processes in production, raw material, transportation, manipulating employees, cleaning, equipment sharing, sub-productions, air particles area, the stock of groceries, production chain, and packaging. Thus, reaching zero risks for cross-contamination is not a viable prospect.

In Brazil, the mandatory use of common names of allergens in warning phrases has been used by the food industries. According to the data presented, Brazil’s preventive labeling is frequently used, and Soybean & products being the most significant insecurity for food choices, present as “May contain” in all Brazilian Pyramid Groups. Soybean is among eight allergenic foods or food groups considered as commonly allergenic on a worldwide basis according to the Codex Alimentarius Commission.^22^ Because of its many uses, soybean avoidance is challenging. However, according to Zurzolo et al.,^23^ this places a significant restriction on food choices and, in some cases, unnecessary.

Ford et al.^24^ found a prevalence of 5.2% of foods containing milk, 2.3% eggs, and 2.2% peanuts due to contamination in the production chain. Such percentages are lower than the results obtained in our sample for these allergens described in the Brazilian food pyramid’s different Groups – the groups Milk & dairy products, Cereals, Sugars, and Fats had high warning percentages for allergens.

On description in food labels, most countries recommend two methods: highlight the presence of an allergen in the list of ingredients itself or use a separate “contains X” declaration for allergenic ingredients.^25^ For PAL, the most common terms were as follows: may contain traces of; produced in a factory which handles; produced on shared equipment which also processes; made in a factory that also produces; not suitable for allergy patients; packed in an environment where; and it may occasionally contain, followed by the common names of foods that cause FAs. In Brazil, the recommendation is only the term “May contain.”^17^

The main limitation of this study was data collection performed exclusively in a given region of the country. Considering Brazil’s continental dimensions, one must consider the differences in products available in the markets according to regional food cultures. Next, local producers with a smaller scale sale were not represented in the studied sample. In contrast, the structuring of the data obtained following the Brazilian food pyramid was relevant to the quality of the information presented, as it guaranteed the coverage of all food groups. Lastly, criteria were used to analyze and qualify the labels based on a federal resolution. The results are unprecedented and provide essential details to the food industry and the regulatory ANVISA agency.^17^ No Brazilian publication was found on this topic.

Despite satisfactory adequacy to Brazil’s resolution, there are benefits of allergic consumers. First, it is necessary to pay attention if the packaging’s information is not hidden by folding the packaging, labels, or twisting (identified in 5% of the sample in this study). This recommendation is essential for supermarket chain professionals who label products.

Information on how to get food safely packaged and manage FAs outside the home is achieved by reading labels correctly.^20,26^ In addition to providing clear and correct information on the food packaging, the allergic patient and the parents/caregivers must know how to identify the information intended for them and interpret it correctly. Food labels also display information about nutrient content and aim to guide healthy food choices. Thus, consumers’ use of this information varies, but it is estimated that about 50% of consumers report reading this information.^27^ The use of nutritional information to shape healthy food choices requires understanding and interpreting the nutrient content and dietary recommendations. Therefore, an individual’s understanding of “knowledge” about what the nutrition label information means should guide the purchase of food.^28^ The analysis presented in this study provided reflections for elaborating an educational material direct food purchase allergic patients. Auxiliary tools such as the “ Food allergy: a manual for reading food labels”^29^ can provide security to risk management in patients’ food choices and facilitate professionals’ educational work. This manual is available at http://bit.ly/publicacao-alergia_alimentar, and/or https://books.apple.com/us/book/alergia-alimentar/id1497950463?ls=1

In conclusion, halthcare professionals who guide patients with FAs can count on good-quality labeling of processed and packaged products. Consequently, they may promote patients’ and parents/caregivers’ education to consult the labels and how to manage the risks, helping to guide the consumption of fresh and minimally processed foods. Regarding PAL, Soybean & products were the most significant insecurity for food choices among Brazilian Pyramid Groups. In addition, soybean is among eight allergenic foods or food groups considered as commonly allergenic on a worldwide basis.^22^
